# The clinical application of flexible bronchoscopy in a neonatal intensive care unit

**DOI:** 10.3389/fped.2022.946579

**Published:** 2022-10-10

**Authors:** Li-qin Ke, Ming-jie Shi, Fei-zhou Zhang, Hu-jun Wu, Lei Wu, Lan-fang Tang

**Affiliations:** ^1^Department of Pulmonology, The Children’s Hospital, Zhejiang University School of Medicine, National Clinical Research Center for Child Health, Hangzhou, Zhejiang, China; ^2^Department of Endoscopy Center, The Children’s Hospital, Zhejiang University School of Medicine, National Clinical Research Center for Child Health, Hangzhou, Zhejiang, China; ^3^Department of Pediatric, The First People’s Hospital of Huzhou, Huzhou, Zhejiang, China

**Keywords:** flexible bronchoscopy, neonates, respiratory diseases, neonatal intensive care unit, laryngomalacia

## Abstract

**Objective:**

Flexible bronchoscopy is widely used in infants and it plays a crucial role. The aim of this study was to investigate the value and clinical safety of flexible bronchoscopy in a neonatal intensive care unit.

**Methods:**

A retrospective analysis was performed on the clinical data of 116 neonates who underwent flexible bronchoscopy and the outcomes of 147 procedures. A correlation analysis was performed on the relationship between flexible bronchoscopy findings, microscopic indications, and clinical disease.

**Results:**

The 147 procedures performed were due to the following reasons: problems related to artificial airways, 58 cases (39.45%); upper respiratory problems, 60 cases (40.81%) (recurrent dyspnea, 23 cases; upper airway obstruction, 17 cases; recurrent stridor, 14 cases; and hoarseness, six cases), lower respiratory problems, 51 cases (34.69%) (persistent pneumonia, 21 cases; suspicious airway anatomical disease, 21 cases; recurrent atelectasis, eight cases; and pneumorrhagia, one case), feeding difficulty three cases (2.04%). The 147 endoscopic examinations were performed for the following reasons: pathological changes, 141 cases (95.92%); laryngomalacia, 78 cases (53.06%); mucosal inflammation/secretions, 64 cases (43.54%); vocal cord paralysis, 29 cases (19.72%); trachea/bronchus stenosis, 17 cases (11.56%) [five cases of congenital annular constriction of the trachea, seven cases of left main tracheal stenosis, one case of the right middle bronchial stenosis, two cases of tracheal compression, and two cases of congenital tracheal stenosis]; subglottic lesions, 15 cases (10.20%) [eight cases of subglottic granulation tissue, six cases of subglottic stenosis, one cases of subglottic hemangioma]; tracheomalacia, 14 cases (9.52%); laryngeal edema, five cases (3.40%); tracheoesophageal fistula, four cases (2.72%); rhinostenosis, three cases (2.04%); tracheal bronchus, three cases (2.04%); glossoptosis, two cases (1.36%); laryngeal cyst, two cases (1.36%); laryngeal cleft, two cases (1.36%); tongue base cysts, one case (0.68%); and pneumorrhagia, one case (0.68%). Complications were rare and mild.

**Conclusion:**

Flexible bronchoscopy is safe and effective for diagnosing and differentiating neonatal respiratory disorders in neonatal intensive care units.

## Introduction

Respiratory disease is a predominantly observed problem in neonatal and pediatric intensive care units (ICUs) (1). It is challenging to explain the complex relationship between infection, lung immaturity, and ventilator-caused lung injury in ventilated patients. This poses a challenge to clinical management. Flexible bronchoscopy (FB) is an important tool for the diagnosis and treatment of various pediatric respiratory diseases (2–4). With the improvement of bronchoscopy equipment and technology, FB has gradually been applied to neonates, especially in the diagnosis and treatment of abnormal airways. The purpose of this study was to investigate flexible bronchoscopic findings and clinical data and discuss the diagnostic contribution and safety of FB in the neonatal ICU (NICU).

## Materials and methods

### Patients

A total of 116 patients underwent FB in the Children’s Hospital, Zhejiang University School of Medicine, NICU, between October 2015 and September 2021. In all, 147 FB procedures were performed in 116 patients. The inclusion criteria were based on the Pediatric Bronchoscopy Guidelines (5); they were as follows: neonates with recurrent pulmonary infection or atelectasis, with recurrent dyspnea or suspicious respiratory tract anomaly; neonates with suspicious tracheal stenosis in the radiological image [X-ray or computed tomography (CT)]; failure to be extubated and difficult intubation; or confirmed congenital esophageal atresia to clarify the presence and position of esophagobronchial fistula before surgery. The exclusion criteria were as follows: severe respiratory diseases with high values of ventilation, multiple organ failure, severe congenital heart disease (CHD) or cardiac dysfunction, coagulopathy, and present hyperthermia.

Data on sex, age on the day of the procedure, gestational age at birth, birth weight, length of NICU stay before FB, indications for bronchoscopy, bronchoscopy findings, and complications were retrospectively collected. All the patients’ parents voluntarily signed the informed consent before FB. This study was approved by the Ethics Committee of the Children’s Hospital affiliated with Zhejiang University School of Medicine, and informed consent was obtained from the child’s parents.

### Bronchoscopy

Flexible bronchoscopy was performed using Olympus (BF-XP 60, BF-XP 260 F, or BF-XP 290) bronchoscopes. To prevent vomiting and aspiration, patients did not consume for 2 h before FB. FB was performed at the bedside in the NICU. Midazolam, injected intravenously at a dose of 0.1 mg/kg, was used for sedation. Local anesthesia with 1% lidocaine was administered. All FBs were performed by two senior pediatric pulmonologists. Depending on the patient’s clinical condition, the bronchoscopy was performed transnasally *via* a laryngeal mask or an endotracheal tube. Stable ventilated newborns were extubated in the process of the procedure so as to evaluate any upper airway anatomic and dynamic disease. Pulse rate, electrocardiogram, and arterial oxygen saturation (SaO_2_) were recorded continuously during the procedure, and non-invasive blood pressure was monitored every 3–5 min. Supplemental oxygen was given by nasal tube on demand. If the SaO_2_ fell below 90%, FB was suspended and attempted again after SaO_2_ recovery.

### Statistical analysis

Data were analyzed using the SPSS Statistics 23.0 software (IBM SPSS Statistics). The values of continuous variables were presented as the mean ± standard deviation. The median and range were presented as non-parametric data. The categorical variables were expressed as quantities and percentages (%). Differences between normally distributed values of two groups were analyzed by an unpaired Student’s *t*-test. Data enumeration was performed using the χ^2^ test and Fisher’s exact probability method. Statistical significance was assumed when *P* < 0.05.

## Results

### General data

A total of 147 FBs were performed in 116 neonates. Among the 116 neonates, 66 were males and 50 were females, with a ratio of 1.32:1. Of 147, 67 (46.58%) were premature babies. At the time of the procedure, the median age ranged from 1 day to 180 days (41.33 ± 36.95 days). Gestational age ranged from 188 days to 291 days (252.18 ± 29.44 days). The birth weight ranged from 750 g to 4,000 g (2,388.68 ± 893.49 g). Concomitant CHD was observed in 104 (70.74%) cases. The median length of NICU stays before FB ranged from 1 day to 180 days (23.87 ± 29.50 days).

The indications for FB were as follows: problems related to artificial airways, 58 cases (39.45%) (extubation failure, 48 cases, and difficulty intubations, 10 cases); upper respiratory problems, 60 cases (40.81%) (recurrent dyspnea, 23 cases; upper airway obstruction, 17 cases; recurrent stridor, 14 cases; and hoarseness, six cases); lower respiratory problems, 51 cases (34.7%) (persistent pneumonia, 21 cases; suspicious airway anatomical disease, 21 cases; recurrent atelectasis, eight cases; and pneumorrhagia, one case); and feeding difficulty, 3 cases (2.04%). The general characteristics of the patients are detailed in Table 1.

**TABLE 1:** General characteristics of the patient (n: 147).

Male, n(%)	66(56.89%)
Female, n(%)	50(43.11%)
Median age(range), days	41.33 ± 36.95(1–180)
Median of gestational age(range), days	252.18 ± 29.44(188–291)
Median of birth weight(range), g	2,388.68 ± 893.49 (750–4,000)
Median length of NICU stay before FB, days	23.87 ± 29.50(1–180)
Premature infants, n(%)	67(45.58%)
Congenital heart diseases, n(%)	104(70.74%)
**Respiratory support before bronchoscopy, n (%)**	
None	12(8.16%)
Oxygen support *via* nasal cannula or mask	10(6.80%)
HFNC	18(12.25%)
NCPAP	26(17.69%)
**Intubation**	81(55.10%)
**FB Indications,** n**(%)**	
***Problems Related With Artificial Airway***	
Extubation failure	48(32.65%)
Difficulty intubations	10(6.80%)
***Upper Respiratory Problems***	
Recurrent dyspnea	23(15.65%)
Upper airway obstruction	17(11.56%)
Recurrent stridor	14(9.52%)
Hoarseness	6(4.08%)
***Lower Respiratory Problems***	
Persistent pneumonia	21(14.29%)
Suspicious airway anatomical disease	21(14.29%)
Recurrent atelectasis	8(5.44%)
Pneumorrhagia	1(0.68%)
***Other***	
Feeding difficulty	3(2.04%)

FB, flexible bronchoscopy; HFNC, high flow nasal cannula; NCPAP, nasal continuous positive airway pressure.

### Results of flexible bronchoscopy

**FIGURE 1:**
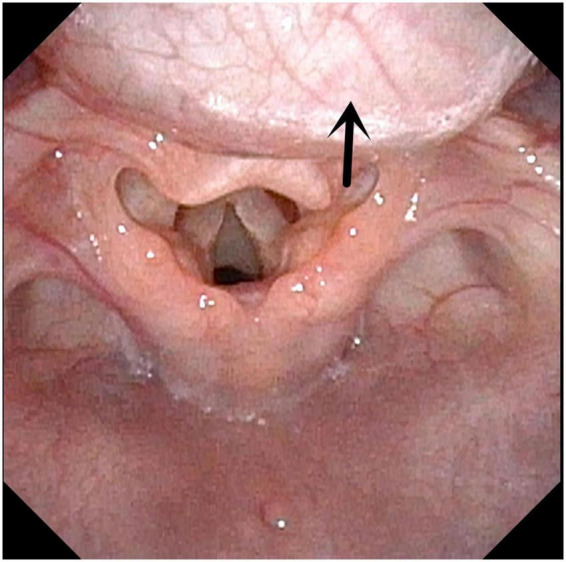
Tongue base cysts. Arrow indicates the Tongue base cysts.

**FIGURE 2:**
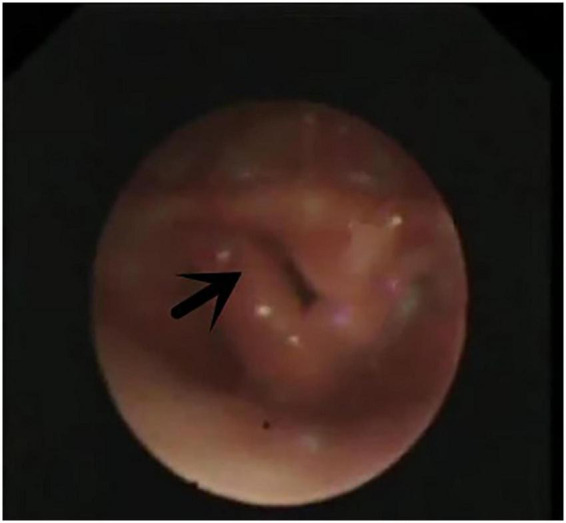
Laryngomalacia. Arrow indicates both sides of epiglottis cartilage curl inward and throat cavity.

**FIGURE 3:**
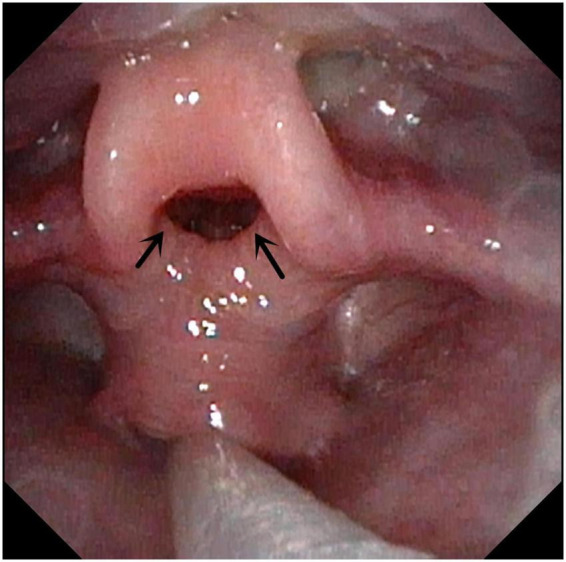
Bilateral vocal cord paralysis. Both arrows indicate the bilateral vocal cord paralysis.

**FIGURE 4:**
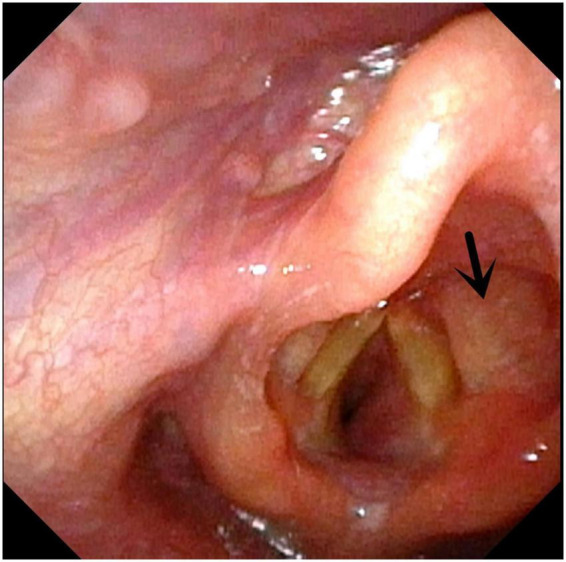
Subglottic hemangioma. Arrow indicates right subglottic hemangioma.

**FIGURE 5:**
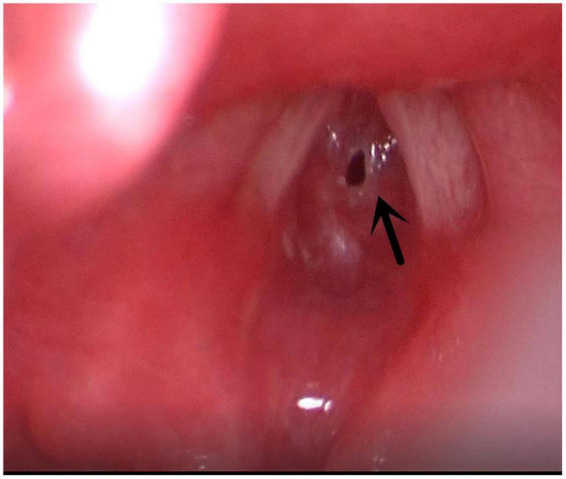
Subglottic stenosis. Arrow indicates subglottic stenosis because of endotracheal intubation.

**FIGURE 6:**
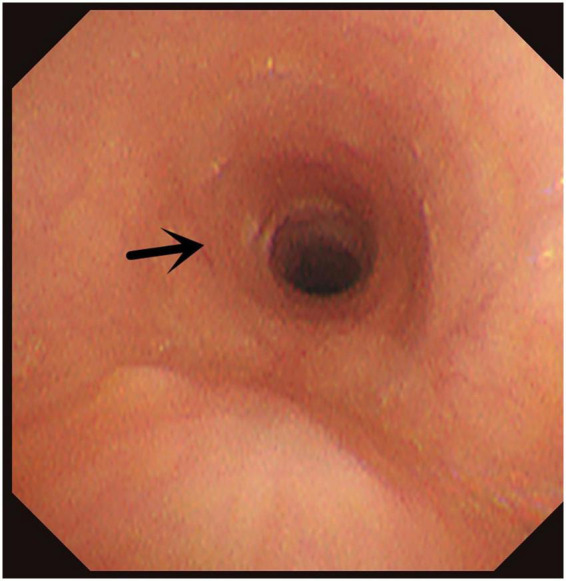
Congenital annular constriction of trachea. Arrow indicates congenital annular constriction of trachea.

Flexible bronchoscopy helped reveal at least one abnormality in 141 cases (95.92%). Among 147 FBs performed, upper respiratory disease accounted for 103 cases. The most common findings were laryngomalacia and vocal cord paralysis (VCP) (53.06% and 19.72% of the patients, respectively). Lower respiratory disease accounted for 71 procedures. Mucosal inflammation/secretions were observed in 64 cases (43.54%), while trachea/bronchus stenosis was observed in 17 cases (11.56%). The biggest proportion of trachea/bronchus stenosis was stenosis of the left main trachea, followed by congenital annular constriction of the trachea. Stenosis of the right middle bronchus, external pressure stenosis of the trachea, and congenital tracheal stenosis were observed in one, two, and two cases, respectively (Figures 1–6 and Tables 2, 3). Both upper and lower respiratory diseases were observed in 32 cases (21.77%), and congenital respiratory malformations were observed in 78 cases (53.06%). In addition, both upper and lower respiratory malformations were observed in 11 cases (7.48%).

**TABLE 2:** The findings of flexible bronchoscopy.

FB findings (147)	N(%)
Normal	6(4.08%)
Upper Respiratory Disease	103(70.06%)
Rhinostenosis	3(2.04%)
Glossoptosis	2(1.36%)
Tongue base cysts	1(0.68%)
Laryngomalacia	78(53.06%)
Laryngeal edema	5(3.40%)
Laryngeal cyst	2(1.36%)
Laryngeal cleft	2(1.36%)
*Vocal cord paralysis*	29(19.72%)
unilateral	11(7.48%)
bilateral	18(12.24%)
*Subglottic lesions*	15(10.20%)
Subglottic granulation tissue	8(5.44%)
Subglottic stenosis	6(4.08%)
Subglottic hemangioma	1(0.68%)
Lower Respiratory Disease	71(48.30%)
Mucosal inflammation/secretions	64(43.54%)
Trachea/bronchus stenosis	17(11.56%)
Congenital Annular Constriction of Trachea	5(3.40%)
Stenosis of the left main trachea	7(4.76%)
Stenosis of the right middle bronchus	1(0.68%)
Tracheal compression	2(1.36%)
Congenital tracheal stenosis	2(1.36%)
Tracheomalacia	14(9.52%)
Tracheoesophageal fistula	4(2.72%)
Tracheal bronchus	3(2.04%)
Pneumorrhagia	1(0.68%)

**TABLE 3:** The indications and findings of FB.

FB indications												
FB findings (n)	Extubation failure	Recurrent dyspnea	Persistent pneumonia	suspicious airway disease	Upper airway obstruction	Recurrent stridor	Difficulty intubations	Recurrent atelectasis	Hoarseness	Feeding difficulty	Pneumorrhagia	total
Rhinostenosis	–	1	–	2	–	–	–	–	–	–	–	3
Glossoptosis	–	1	–	–	–	1	1	–	–	–	–	3
Tongue base cysts		1										1
Laryngomalacia	13	14	6	12	10	9	5	3	4	2	–	78
Laryngeal edema	1	1	–	–	2	–	1	–	–	–	–	5
Laryngeal cyst	–	–	–	–	2	–	–	–	–	–	–	2
Laryngeal cleft	–	–	–	1	–	–	–	–	–	1	–	2
Vocal cord paralysis	3	7	–	7	3	4	–	–	4	1	–	29
Unilateral	1	3	–	3	–	2	–	–	2	–	–	11
Bilateral	2	4	–	4	3	2	–	–	2	1	–	18
Subglottic granulation tissue	4	1	–	2	1	–	–	–	–	–	–	8
Subglottic stenosis	–	1	–	–	–	1	4	–	–	–	–	6
Subglottic hemangioma	1	–	–	–	–	–	–	–	–	–	–	1
Mucosal inflammation/secretions	20	9	21	3	1	–	2	8	–	–	–	64
Congenital annular constriction of trachea	–	1	–	3	–	–	1	–	–	–	–	5
Stenosis of the left main trachea	2	–	1	2	–	–	–	2	–	–	–	7
Stenosis of the right middle bronchus	–	–	–	1	–	–	–	–	–	–	–	1
Tracheal compression	1	–	–	1	–	–	–	–	–	–	–	2
Congenital tracheal stenosis	–	–	–	–	1	–	1	–	–	–	–	2
Tracheomalacia	6	2	1	–	1	1	1	2	–	–	–	14
Tracheoesophageal fistula	–	–	3	–	–	–	–	–	–	1	–	4
Tracheal bronchus	2	1	–	–	–	–	–	–	–	–	–	3
Pneumorrhagia	–	–	–	–	–	–	–	–	–	–	1	1

### Correlations

The incidence of laryngomalacia was higher in newborns with CHD than in those without CHD (58.65% vs. 37.78%, *P* = 0.046). While the incidence of subglottic lesions, mucosal inflammation/secretions, trachea/bronchus stenosis, and tracheomalacia was higher, there was no statistical difference. The length of stay did not significantly differ between the two groups. The prevalence rates of laryngomalacia, vocal cord paralysis, and trachea/bronchus stenosis were significantly higher among premature infants. Mature infants had a significantly shorter length of hospital stay. The correlations between the FB findings and other diseases are presented in Table 4.

**TABLE 4:** The correlations between CHD and non–CHD, premature and mature.

FB Finding	CHD vs. non-CHD	χ^2^/Z value	*P*	Premature vs. Mature	χ^2^/Z value	*P*
Rhinostenosis	2(1.92%) vs. 1(2.33%)	0.025	1.000	2(2.99%) vs. 1(1.25%)	0.549	0.459
Glossoptosis	3(2.88%) vs. 0(0%)	1.266	0.556	0(0%) vs. 3(3.75%)	2.565	0.310
Laryngomalacia	61(58.65%) vs. 17(37.78%)	4.465	0.035*	45(67.16%) vs. 33(41.25%)	9.831	0.002*
Laryngeal edema	4(3.84%) vs. 1(2.33%)	0.214	1.000	2(2.99%) vs. 3(3.75%)	0.065	1.000
Laryngeal cyst	1(0.96%) vs. 1(2.33%)	0.422	0.501	2(2.99%) vs. 0(0%)	2.412	0.206
Laryngeal cleft	2(1.92%) vs. 0(0%)	0.838	1.000	2(2.99%) vs. 0(0%)	2.412	0.206
Vocal cord paralysis	19(18.27%) vs. 10(23.26%)	0.478	0.501	8(11.94%) vs. 21(26.25%)	4.715	0.030*
Subglottic lesions	12(11.53%) vs. 4(9.30%)	0.157	0.916	7(10.44%) vs. 9(11.25%)	0.024	0.876
Mucosal inflammation/secretions	50(48.08%) vs. 14(32.56%)	2.980	0.084	29(43.28%) vs. 35(43.75%)	0.003	0.955
Trachea/bronchus stenosis	14(13.46%) vs. 3(6.98%)	1.251	0.404	10(14.95%) vs. 7(8.75%)	1.360	0.244
Tracheomalacia	12(11.53%) vs. 2(4.65%)	1.675	0.324	11(16.42%) vs. 3(3.75%)	6.791	0.009*
Tracheoesophageal fistula	2(1.92%) vs. 2(4.65%)	0.855	0.581	0(0%) vs. 4(5.00%)	3.444	0.178
Length of NICU stay[day, median(range)]	10.50(1,180) vs. 15.00(1,130)	−0.657	0.511	38.00(1,180) vs. 17.00(1,90)	−3.572	0.000*

CHD, congenital heart diseases; FB, flexible bronchoscopy; NICU, neonatal intensive care unit.

*Means the difference is statistically significant.

### Complications

In 35 (23.8%) procedures, mild hypoxemia (80% < SaO_2_ < 90%) was observed. One case had bradycardia and severe hypoxemia (approximately 60%) during the bronchoscopy. After a brief pause, oxygen suction was provided. The patient quickly returned to his baseline respiratory support following the procedure. No significant complications, such as severe airway trauma, pneumothorax, serious hemorrhage, shock, or death, occurred during the procedures.

## Discussion

The incidence of respiratory diseases in neonates is relatively high and is responsible for most neonatal hospitalizations. Over the years, due to its diagnostic and therapeutic value, FB has grown quickly in pediatrics (5, 6). In addition, it has a high safety profile. A regular radiological examination cannot give a confirmatory diagnosis (7). FB serves as an alternative way to diagnose anomalies of the respiratory tract in such patients. FB helps diagnose many unexplained lung problems that cannot be dealt with clinical regular examinations and treatment (8). Several studies have reported the contribution of FB in the diagnosis and therapeutic process in different patient groups. In a review that involved 27 pediatric studies, Ridley et al. reported that FB helped diagnose 82% of the patients (9). This rate was even higher among patients with suspected airway disease and those who were dependent on ventilators (10). Our results also emphasize the high diagnostic contribution of FB.

Mechanical ventilation has mostly been proven to be very important for the survival of extremely premature neonates and continues to play a major role in NICU (11, 12). However, ventilator dependence is an important issue in these patients, and FB helps evaluate the airways and develop necessary interventions (13, 14). The most common indication for bronchoscopy in our study was extubation failure, accounting for 32.65% of the patients. Two other significant indications for FB in newborns are recurrent dyspnea and suspicious airway disease. The largest series reporting on neonatal FB included 599 procedures, and the most common indications were nosocomial pneumonia (28.2%), ventilator dependence (13.3%), and unilateral lung disease (13.3%) (15).

Other common indications for FB in our study were pneumonia and atelectasis. Previous studies have reported a rate of 22–77% for pneumonia and atelectasis as indications for FB (15–17). Due to the anatomical features of the airways, excessive airway secretions, and lack of secondary surfactants, newborns are prone to atelectasis. FB helps determine the underlying cause of persistent pneumonia and recurrent atelectasis (18).

Our study reported that FB revealed at least one positive finding in 95.92% of the patients admitted to NICU. Previous research reported that 79–98% of patients in the NICU have at least one abnormality detected using FB (16, 19, 20). Herein, the high rate of abnormal findings may be secondary to infants’ reluctance to perform early FB, resulting in severe and persistent symptoms that precede FB.

Similar to the findings of previous studies, respiratory malformation was the most common finding of FB (6, 21). The most common upper airway malformations in our study were laryngomalacia and glottis dysplasia. Tracheal stenosis, followed by tracheomalacia, is the most common lower airway abnormality. FB is recognized as an excellent diagnostic tool for laryngomalacia (22, 23). Our study revealed that laryngomalacia was the most common cause of upper airway obstruction, recurrent dyspnea, stridor, and hoarseness in newborns. In a retrospective report of 196 bronchoscopies, it was documented that airway malacia was found in 47.4% of cases (19). In addition, a 60–70% incidence of laryngomalacia was reported in neonates and children with stridor (24). About 51.7% of infants with laryngomalacia have developed secondary airway lesions, with subglottic stenosis and tracheomalacia being the most common lesions (25). In our study, subglottic stenosis and tracheomalacia occurred in 4.08% and 4.76% of the neonates with laryngomalacia, respectively.

Vocal cord paralysis accounts for the second most frequent laryngeal anomaly among infants. VCP is often manifested as stridor, hoarseness, respiratory distress, weak crying or dysphagia, and repeated aspiration pneumonia, which leads to growth and development disorders (26, 27). This study finds that VCP occurs with recurrent dyspnea, suspicious airway disease, stridor, hoarseness, difficulty intubations, extubation failure, and feeding difficulty. A similar distribution of bilateral (48%) and unilateral (52%) VCP was reported by a retrospective chart review of 102 VCP cases (28). The left recurrent laryngeal nerve is positioned lower and follows a longer path; therefore, the probability of damage to the left recurrent laryngeal nerve is much higher than that to the right recurrent laryngeal nerve (29). This finding was confirmed by our study (left VCP, 10 cases, and right VCP, one case), where the right VCP may have occurred due to nerve compression by the right laryngeal cyst.

In our study, the second most common finding was mucosal inflammation/secretions, particularly in neonates with extubation failure, pneumonia, and atelectasis. Bronchial mucosa swelling and hyperemia, bronchial inflammation, purulent secretions, and other bronchial mucosa inflammatory changes were revealed during endoscopy (30). This might be related to cough weakness, severe lung infection with hypersecretion, nervous system and/or muscle disease, muscle relaxants, sedatives, or the use of anesthesia after surgery or mechanical ventilation. Moreover, ventilator dependence may lead to ventilator-associated lung disease and ventilator-associated pneumonia (11, 31).

It has been reported that children with CHD may be accompanied by complications of airway malformation (32, 33). A prospective cohort study of 30 infants found significant airway narrowing in 50% of patients treated with FB (34). Billings et al. (35) reported that 30.2% of children with CHD and obstructive respiratory disease were diagnosed with tracheobronchomalacia and tracheal stenosis during FB. Long-term intubation was more commonly required in neonates with CHD. In this study, pulmonary artery sling along with tracheal stenosis was observed in one patient. Left main bronchial stenosis was observed in three patients with atrial septal defect, ventricular septal defect, and patent ductus arteriosus. One patient with pulmonary valve stenosis had left main bronchial stenosis. Chest CT needs to be performed before surgery in patients with abnormal cardiovascular disease to confirm the presence of suspected respiratory malformations (36, 37).

Some airway malformations may still go unnoticed even though multislice spiral CT can perform three-dimensional vascular and airway reconstruction to assess airway anatomy. Airway anatomy and airflow dynamics changes can be observed by FB under direct vision, thereby compensating for the defects of multi-slice spiral CT (7). Herein, CT and endoscopy helped diagnose eight and 18 cases of lower airway abnormalities, confirmed by CT, respectively. Therefore, preoperative pulmonary CT and FB examinations can be performed simultaneously in patients with CHD to better evaluate airway function, reduce perioperative respiratory complications, and improve the postoperative survival rate.

Common clinical complications of FB include nasal trauma and epistaxis, laryngeal spasm, laryngeal edema, cough and/or bronchospasm, pneumothorax or mediastinal emphysema, hemorrhage, hypoxemia, and fever and infections (5). FB has been reported to be safe, with no surgery-related mortality in pediatric ICU and NICU (19, 35, 38). Minimal complications were reported in our study (transient hypoxemia in 35 neonates), supporting the findings that FB is a safe procedure when performed by experienced operators under proper monitoring.

There are some limitations to our study. It may involve some repeated flexible bronchoscopies in the same patients, which may cause the same repeated results. Additionally, as it is a retrospective study, we could not assess the prognosis in detail.

## Conclusion

Flexible bronchoscopy plays an important role in diagnosing and differentiating neonatal respiratory diseases. FB is relatively safe in the NICU, with a rare occurrence of serious complications.

## Data availability statement

The original contributions presented in this study are included in the article/supplementary material, further inquiries can be directed to the corresponding authors.

## Ethics statement

The studies involving human participants were reviewed and approved by the Children’s Hospital Affiliated to Zhejiang University School of Medicine. Written informed consent to participate in this study was provided by the participants’ legal guardian/next of kin.

## Author contributions

L-QK and M-JS completed the first draft. F-ZZ and H-JW participated in the data collection and improved the later revision of the article. LW and L-FT revised the manuscript to ensure its authenticity and practicability. All authors approved the final manuscript as submitted and agreed to be accountable for all aspects of the work.

## Abbreviations

CHD: congenital heart diseases
CT: computed tomography
FB: flexible bronchoscopy
HFNC: high flow nasal cannula
ICU: intensive care units
NCPAP: nasal continuous positive airway pressure
NICU: neonatal intensive care unit
SaO_2_: arterial oxygen saturation
VCP: vocal cord paralysis.

## References

[1] Divecha C Tullu MS Chaudhary S. Burden of respiratory illnesses in pediatric intensive care unit and predictors of mortality: experience from a low resource country. *Pediatr Pulmonol.* (2019) 54:1234–41. 10.1002/ppul.24351 31087783

[2] Estella A. [Analysis of 208 flexible bronchoscopies performed in an intensive care unit]. *Med Intensiva.* (2012) 36:396–401. 22192316 10.1016/j.medin.2011.11.005

[3] Schramm D Yu Y Wiemers A Vossen C Snijders D Krivec U Pediatric flexible and rigid bronchoscopy in European centers-availability and current practice. *Pediatr Pulmonol.* (2017) 52:1502–8. 10.1002/ppul.23823 28910517

[4] Ndilanha DA Shayo GA Hassan R Byomuganyizi M Lema LEK. Diagnoses from lung specimen collected through flexible bronchoscopy from patients in a tertiary hospital in dar es salaam tanzania: a retrospective cross-sectional study. *BMC Pulm Med.* (2019) 19:214. 10.1186/s12890-019-0972-x PMC6854817 31727025

[5] Pérez-Frías J Moreno Galdó A Pérez Ruiz E Barrio Gómez De Agüero MI Escribano Montaner A Caro Aguilera P. [Pediatric bronchoscopy guidelines]. *Arch Bronconeumol.* (2011) 47:350–60. 21600686 10.1016/j.arbres.2011.04.003

[6] Hysinger EB Hart CK Burg G De Alarcon A Benscoter D. Differences in flexible and rigid bronchoscopy for assessment of tracheomalacia. *Laryngoscope.* (2021) 131:201–4. 32282085 10.1002/lary.28656

[7] Soyer T. The role bronchoscopy in the diagnosis of airway disease in children. *J Thorac Dis.* (2016) 8:3420–6. 28066622 10.21037/jtd.2016.11.87 PMC5179461

[8] Kohelet D Arbel E Shinwell ES. Flexible fiberoptic bronchoscopy–a bedside technique for neonatologists. *J Matern Fetal Neonatal Med.* (2011) 24:531–5. 10.3109/14767058.2010.501123 20617894

[9] Field-Ridley A Sethi V Murthi S Nandalike K Li ST. Utility of flexible fiberoptic bronchoscopy for critically ill pediatric patients: a systematic review. *World J Crit Care Med.* (2015) 4:77–88. 10.5492/wjccm.v4.i1.77 25685726 PMC4326767

[10] Terkawi RS Altirkawi KA Terkawi AS Mukhtar G Al-Shamrani A. Flexible bronchoscopy in children: Utility and complications. *Int J Pediatr Adolesc Med.* (2016) 3:18–27. 30805463 10.1016/j.ijpam.2015.12.003 PMC6372410

[11] Dalgleish S Kostecky L Charania I. Special considerations in neonatal mechanical ventilation. *Crit Care Nurs Clin North Am.* (2016) 28:477–98. 28236394 10.1016/j.cnc.2016.07.007

[12] Bhat JI Charoo BA Zahoor S Ahmad QI Ahangar AA. Role of flexible bronchoscopy in ventilator-dependent neonates. *Indian Pediatr.* (2020) 57:922–5. 33089808 10.1007/s13312-020-1996-2 PMC7605484

[13] Pereira KD Smith SL Henry M. Failed extubation in the neonatal intensive care unit. *Int J Pediatr Otorhinolaryngol.* (2007) 71:1763–6. 17850890 10.1016/j.ijporl.2007.07.018

[14] Ferguson KN Roberts CT Manley BJ Davis PG. Interventions to improve rates of successful extubation in preterm infants: a systematic review and meta-analysis. *JAMA Pediatr.* (2017) 171:165–74. 27918754 10.1001/jamapediatrics.2016.3015

[15] Mackanjee HR Naidoo L Ramkaran P Sartorius B Chuturgoon AA. Neonatal bronchoscopy: role in respiratory disease of the newborn-A 7 year experience. *Pediatr Pulmonol.* (2019) 54:415–20. 10.1002/ppul.24243 30620142

[16] Manna SS Durward A Moganasundram S Tibby SM Murdoch IA. Retrospective evaluation of a paediatric intensivist-led flexible bronchoscopy service. *Int Care Med.* (2006) 32:2026–33. 10.1007/s00134-006-0351-y 16941167

[17] Tang LF Xu YC Wang YS Wang CF Zhu GH Bao XE Airway foreign body removal by flexible bronchoscopy: experience with 1027 children during 2000-2008. *World J Pediatr.* (2009) 5:191–5. 10.1007/s12519-009-0036-z 19693462

[18] Gokdemir Y Cakir E Kut A Erdem E Karadag B Ersu R Bronchoscopic evaluation of unexplained recurrent and persistent pneumonia in children. *J Paediatr Child Health.* (2013) 49:E204–7. 10.1111/jpc.12124 23438344

[19] Atag E Unal F Yazan H Girit S Uyan ZS Ergenekon AP Pediatric flexible bronchoscopy in the intensive care unit: a multicenter study. *Pediatr Pulmonol.* (2021) 56:2925–31. 34236776 10.1002/ppul.25566

[20] Davidson MG Coutts J Bell G. Flexible bronchoscopy in pediatric intensive care. *Pediatr Pulmonol.* (2008) 43:1188–92. 19009620 10.1002/ppul.20910

[21] Choi J Dharmarajan H Yu J Dunsky KA Vece TJ Chiou EH Diagnostic flexible versus rigid bronchoscopy for the assessment of tracheomalacia in children. *J Laryngol Otol.* (2018) 132:1083–7. 10.1017/S0022215118002050 30565533

[22] Parkes WJ Propst EJ. Advances in the diagnosis, management, and treatment of neonates with laryngeal disorders. *Semin Fetal Neonatal Med.* (2016) 21:270–6. 27049674 10.1016/j.siny.2016.03.003

[23] Thorne MC Garetz SL. Laryngomalacia: review and Summary of current clinical practice in 2015. *Paediatr Respir Rev.* (2016) 17:3–8. 10.1016/j.prrv.2015.02.002 25802018

[24] Daniel SJ. The upper airway: congenital malformations. *Paediatr Respir Rev.* (2006) 7:S260–3. 16798587 10.1016/j.prrv.2006.04.227

[25] Dickson JM Richter GT Meinzen-Derr J Rutter MJ Thompson DM. Secondary airway lesions in infants with laryngomalacia. *Ann Otol Rhinol Laryngol.* (2009) 118:37–43. 19244962 10.1177/000348940911800107

[26] Ryan MA Upchurch PA Senekki-Florent P. Neonatal vocal fold paralysis. *Neoreviews.* (2020) 21:e308–22. 32358144 10.1542/neo.21-5-e308

[27] Erdem E Gokdemir Y Unal F Ersu R Karadag B Karakoc F. Flexible bronchoscopy as a valuable tool in the evaluation of infants with stridor. *Eur Arch Otorhinol.* (2013) 270:21–5. 10.1007/s00405-012-2057-9 22639201

[28] Daya H Hosni A Bejar-Solar I Evans JN Bailey CM. Pediatric vocal fold paralysis: a long-term retrospective study. *Arch Otolaryngol Head Neck Surg.* (2000) 126:21–5. 10628706 10.1001/archotol.126.1.21

[29] Ada M Isildak H Saritzali G. Congenital vocal cord paralysis. *J Craniofac Surg.* (2010) 21:273–4. 20098198 10.1097/SCS.0b013e3181c5a456

[30] An SH Wang MM Li JY Zheng BJ Wang YY Zhao QJ [Role of flexible bronchoscopy in the diagnosis and treatment of refractory pneumonia in children]. *Zhongguo Dang Dai Er Ke Za Zhi.* (2011) 13:547–50. 21752319

[31] Saydain G. Ventilator-associated pneumonia in advanced lung disease: a wakeup call. *Lung India.* (2014) 31:1–3. 10.4103/0970-2113.125884 24669073 PMC3960802

[32] de Trey LA Dudley J Ismail-Koch H Durward A Bellsham-Revell H Blaney S Treatment of severe tracheobronchomalacia: ten-year experience. *Int J Pediatr Otorhinolaryngol.* (2016) 83:57–62. 10.1016/j.ijporl.2016.01.022 26968054

[33] Lee YS Jeng MJ Tsao PC Soong WJ Chou P. Prognosis and risk factors for congenital airway anomalies in children with congenital heart disease: a nationwide population-based study in taiwan. *PLoS One.* (2015) 10:e0137437. 10.1371/journal.pone.0137437 PMC4559478 26334302

[34] Nayak PP Sheth J Cox PN Davidson L Forte V Manlhiot C Predictive value of bronchoscopy after infant cardiac surgery: a prospective study. *Int Care Med.* (2012) 38:1851–7. 10.1007/s00134-012-2702-1 23011533

[35] Billings KR Rastatter JC Lertsburapa K Schroeder JW Jr. An analysis of common indications for bronchoscopy in neonates and findings over a 10-year period. *JAMA Otolaryngol Head Neck Surg.* (2015) 141:112–9. 10.1001/jamaoto.2014.3198 25521999

[36] Singhal M Gupta P Singh RS Rohit MK Sodhi KS Khandelwal N. Cardiovascular causes of pediatric airway compression: a pictorial review. *Curr Probl Diagn Radiol.* (2015) 44:505–10. 10.1067/j.cpradiol.2015.04.005 25998073

[37] Zhang AM Xu J Wang JM Huang FR Zhong LL Lin L [Application of fiberoptic bronchoscopy in the diagnosis and treatment of neonatal respiratory diseases]. *Zhongguo Dang Dai Er Ke Za Zhi.* (2014) 16:306–8. 24661527

[38] Yan C Hu Y Qiu G Gong X Elda D. The clinical safety and efficacy of flexible bronchoscopy in a neonatal intensive care unit. *Exp Ther Med.* (2020) 20:95. 10.3892/etm.2020.9223 32973944 PMC7507083

